# BRAIN NETWORKS AND FUNCTIONAL RESERVE IN THE PRESENCE OF AMYLOID PATHOLOGY

**DOI:** 10.1093/geroni/igae098.0150

**Published:** 2024-12-31

**Authors:** Paul Laurienti

**Affiliations:** Wake Forest University School of Medicine, Winston-Salem, North Carolina, United States

## Abstract

Background: Amyloid beta (Aβ) is a biomarker of Alzheimer’s disease and has been linked to cognitive and physical function decline. However, significant inter-individual variability exists with respect to the influence of Aβ on cognitive and motoric outcomes. The current study examined the potential contribution of brain networks on this inter-individual variability. Methods: Community-dwelling older adults (n=81, mean age=76ys, %female=51) from a larger longitudinal brain network study were included. Participants were adjudicated as cognitively normal at baseline. Each had resting-state functional magnetic resonance and Aβ positron emission tomography scans. A statistical interaction model was used to determine if network integrity moderated the effects of global Aβ on physical function as measured with the expanded short physical performance battery (eSPPB). Results: At baseline the central executive network (CEN) and the basal ganglia network (BGN) significantly (p<0.05) moderated Aβ associations with eSPPB. Physical function was highest in those individuals with high network integrity and low Aβ. Function was lowest in those with both low network integrity and high Aβ. Neither Aβ-network interaction was significant for longitudinal eSPPB change scores. Conclusion: Integrity in the CEN and BGN may underlie reserve in the presence of Aβ pathology as those individuals with high network integrity maintained their physical function. Given that these brain networks exhibit plasticity, they are a potential intervention target in older adults exhibiting functional decline in the presence of Aβ. Future work will need to replicate the findings in a larger and more diverse sample of older adults.

